# Mammographic density, lobular involution, and risk of breast cancer

**DOI:** 10.1038/sj.bjc.6604635

**Published:** 2008-09-09

**Authors:** O M Ginsburg, L J Martin, N F Boyd

**Affiliations:** 1 Campbell Family Institute for Breast Cancer Research, Ontario Cancer Institute, Toronto, Canada

**Keywords:** mammographic density, involution, breast cancer, risk

## Abstract

In this review, we propose that age-related changes in mammographic density and breast tissue involution are closely related phenomena, and consider their potential relevance to the aetiology of breast cancer. We propose that the reduction in mammographic density that occurs with increasing age, parity and menopause reflects the involution of breast tissue. We further propose that age-related changes in both mammographic density and breast tissue composition are observable and measurable phenomena that resemble Pike's theoretical construct of ‘breast tissue ageing’. Extensive mammographic density and delayed breast involution are both associated with an increased risk of breast cancer and are consistent with the hypothesis of the Pike model that cumulative exposure of breast tissue to hormones and growth factors that stimulate cell division, as well as the accumulation of genetic damage in breast cells, are major determinants of breast cancer incidence.

Henson and Tarone (1994) proposed that variations in the rate or extent of the decrease in the number and size of breast lobules with increasing age, referred to as breast tissue involution, might be an important factor in the aetiology of breast cancer. They also drew attention to similarities between the histological changes of involution and changes in radiological features of the breast, which were then described in terms of Wolfe's patterns and are now usually referred to as ‘mammographic density’.

Since then, there have been advances in our understanding of the relationship between both breast histology and mammographic density to the risk of breast cancer, and of the factors that influence these features. Vachon *et al* (2007a) recently drew attention to similarities between age-related breast involution and mammographic density.

In this review, we discuss some of the literature related to mammographic density, breast tissue involution, and breast cancer risk. We propose that age-related changes in mammographic density and breast involution are closely related phenomena, and consider their potential relevance to the aetiology of breast cancer in the light of the Pike model of mammary carcinogenesis.

## Mammographic density and breast cancer risk

The radiographic appearance of the breast varies among women of the same age because of variations in breast tissue composition (see further in ‘Age and breast tissue composition’ section below) and the X-ray attenuation properties of the different types of tissue (Johns and Yaffe, 1987). Fat attenuates X-rays least and appears dark on a mammogram, whereas stroma and epithelium attenuate X-rays more and appear white.

Wolfe (1976a, 1976b) related these variations in the appearance of the mammogram to the risk of breast cancer using a qualitative classification with four categories: N1 for breast tissue comprising mainly fat; P1 and P2 for the appearance of ductal prominence of less than 25% or greater than 25%, respectively, or more of the breast area; and DY extensive ‘dysplasia’. Subsequently, the American College of Radiology introduced the BI-RADS classification with four categories: (1) almost entirely fat (low density); (2) scattered fibroglandular densities (average density); (3) heterogeneously dense (high density); and (4) extremely dense (very high density) (American College of Radiology (ACR), 2003). Most well-designed epidemiological studies have found that Wolfe's classification and the BI-RADS system do identify individuals at different risks for breast cancer (Saftlas and Szklo, 1987; Oza and Boyd, 1993; Ziv *et al*, 2004), but quantitative methods that measure and express the area of dense tissue as a percent of the area of the breast in the image have, in general, given more consistent results and created larger gradients of risk (Warner *et al*, 1992; McCormack and dos Santos Silva, 2006).

As reviewed recently by McCormack and dos Santos Silva (2006), at least 15 independent studies that used quantitative methods to assess mammographic density (10 case–control studies and 5 cohort studies) with a total of 6274 cases of breast cancer and 11 638 controls have been published to date. Although methods of measurement and definitions of density vary among studies, women with density in more than 60–75% of the breast have consistently been found to have a four- to six-fold greater risk of breast cancer than women with little or no density. Risk estimates were larger in studies that used percent density rather than Wolfe or BI-RADS classifications, and associations between density and risk were 20–30% stronger in studies with incident rather than prevalent cancers.

Extensive breast density is therefore one of the strongest known risk factors for developing breast cancer, second only to age and carrying a BRCA1 or BRCA2 mutation. Although less than 5% of unselected breast cancer patients have a BRCA1 or BRCA2 mutation, extensive mammographic density is common among women with breast cancer, and estimates of attributable risk show that densities in more than 50% of the breast may account for about a third of all breast cancers (Byrne *et al*, 1995; Boyd *et al*, 2007).

A previous study has shown that extensive mammographic density is also strongly associated with the risk of benign breast disease. Compared to women with no density, risk of hyperplasia without atypia was increased 12.2-fold (95% CI: 2.97–50.14), and that of atypical hyperplasia and *in situ* breast cancer increased 9.7-fold (95% CI: 1.75–53.97) in women with density in more than 75% of the mammogram (Boyd *et al*, 1992).

**Figure 1:**
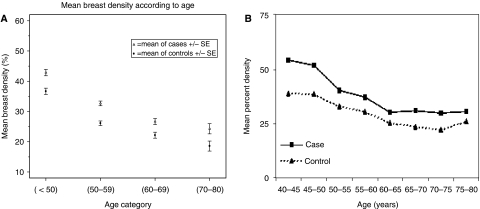
Percent mammographic density *vs* age. (**A**) Baseline percentage mammographic density in women from three mammographic screening programmes. Reproduced with kind permission of BioMed Central from Martin and Boyd, 2008. (**B**) Unadjusted mean percent density as a function of age group and case status. Reproduced with kind permission of American Association for Cancer Research from Maskarinec *et al*, 2006.

Percent mammographic density is less extensive in parous than in nulliparous women and in postmenopausal than premenopausal women, and it is inversely associated with body weight (reviewed by Boyd *et al*, 2005). Cross-sectional and longitudinal studies show that average percent mammographic density declines with increasing age (Figure 1) (Maskarinec *et al*, 2006; Martin and Boyd, 2008). This may seem paradoxical as breast cancer risk increases with age. However, we have proposed that this apparent paradox can be resolved by reference to the concept of ‘breast tissue age’ described by Pike and discussed in the next section.

## Age and breast cancer incidence

**Figure 2:**
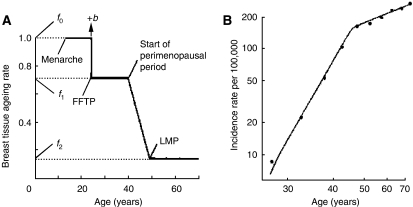
Breast tissue ageing (**A**) and the age-specific incidence of breast cancer (**B**). Reproduced with kind permission of Nature Publishing Group from Pike *et al*, 1983.

In 1983, Pike proposed a model that accounted for the effect of the principal reproductive and endocrine risk factors for breast cancer on the incidence of the disease. The model is based on the concept that the rate of ‘breast tissue ageing’, rather than chronological age, is the relevant measure for describing the effects of ‘hormonal’ risk factors on the age-specific incidence of breast cancer (Pike *et al*, 1983). The concept of ‘breast tissue ageing’ is related to the effects of hormones on the kinetics of breast cells and the associated accumulation of genetic damage. As shown in Figure 2A, the rate of ‘breast tissue ageing’ is most rapid at the time of menarche, slows with each pregnancy, slows further in the perimenopausal period, and is least after the menopause. After fitting suitable numerical values for these parameters, Pike showed that cumulative exposure to ‘breast tissue ageing’, given by the area under the curve in Figure 2A, described the age-incidence curve for breast cancer in the United States, shown in Figure 2B. The general properties of the model have since been confirmed by observation when applied to the Nurses Health Study by Rosner and Colditz (1996), who extended the model initially to include the effects of number and spacing of pregnancies and subsequently to include other non-reproductive risk factors.

## Age and breast tissue composition

Increasing age is associated with a reduction in glandular tissue and an increase in fat in the breast. For example, Gertig *et al* (1999) found in surgical biopsies sampled from the Nurses Health Study that the proportion of the biopsy occupied by both the epithelium and stroma deceased with increasing age at biopsy. This tissue was, however, taken from regions of the breast known or suspected to contain disease.

Breast tissue not sampled from sites of breast disease was obtained by Bartow *et al* using forensic autopsy material from 519 women examined in the New Mexico Office of the Medical Investigator between 1978 and 1983. After subcutaneous mastectomy, whole breast mammograms were obtained and the specimens sectioned at 1 cm intervals. Tissue slices were examined with high-dose non-screen radiography, and random samples were taken for histological examination (Bartow *et al*, 1987). Using this material, Li *et al* (2005) carried out a histological evaluation of 236 subjects, sampled according to mammographic density and age. Quantitative microscopy was used to measure both the nuclear area of epithelial and non-epithelial cells and the areas of glandular tissue and collagen. Details of the methods used are given elsewhere.

**Figure 3:**
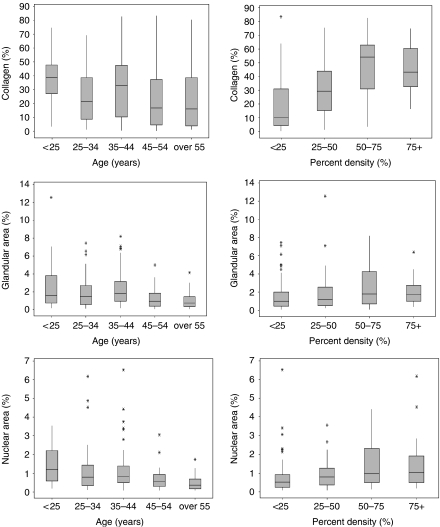
Boxplots of histological measures *vs* age (left hand panels); and histological measures *vs* percent density (right hand panels), data from Boyd and colleagues (unpublished).

Statistically significant inverse associations were found between age and each of the breast tissue measurements (Figure 3). These associations were not changed by adjustment for weight or BMI, except percent collagen, where the inverse association with age became stronger after adjustment (data not shown).

The study of Li *et al* (2005) showed an association between each of the breast tissue features measured and percent mammographic density in the image of the tissue slice from which the sample was taken (Figure 3 right-hand panels). Greater percent density was statistically significantly associated with a greater proportion of collagen, glandular structures, and total nuclear area (as well as epithelial and non-epithelial nuclear area, data not shown). The risk factors that were found on univariate analysis (adjusted for age) to be associated with percent density were in general also associated, in the same direction, with measures of breast tissue (see Li *et al*, 2005). Age, body mass index, and postmenopausal status were significantly and inversely associated with mammographic density and all of the breast tissue measurements. Weight was also negatively associated with all of the tissue measurements, except for glandular area. Parity and number of births were associated inversely only with percent collagen.

## Breast tissue composition and risk of breast cancer

The association between the type of tissue in the breast and the risk of breast cancer has been described extensively. Women diagnosed with carcinoma *in situ* and atypical hyperplasia or hyperplasia without atypia have an approximately 9.4, and 1.5 times, respectively, greater risk of developing invasive breast cancer when compared to the general population of women of the same age (Dupont and Page, 1985; Hartmann *et al*, 2005).

More recently, a quantitative classification of breast tissue has been shown to be related to breast cancer risk. Milanese *et al* (2006) examined the risk of breast cancer associated with variations in lobular involution in the Mayo Benign Breast Disease Cohort of 8736 women with benign breast disease who had biopsies between 1967 and 1991, and who had subsequently been followed for a median of 17 years. All slides were reviewed by a breast pathologist ‘blinded’ to the patient's age, cancer outcome, or original histological diagnosis. Involution in the biopsy was classified into one of three categories according to the extent of lobular involution in the terminal duct lobular units (TDLUs) in the background tissue as: none (0% TDLUs involuted), partial (1–74% TDLUs involuted), or complete (⩾75% TDLUs involuted).

Complete lobular involution was present in 5.8% of women aged 40–49 years and in 21.6% of women aged 50–59 years, and the degree of involution was greater in nulliparous than in parous women. (As noted above, parity has been found in the study of Li *et al* to be inversely associated with collagen but not with other tissue components–see ‘Age and breast tissue composition’ section.)

Risk of breast cancer associated with variations in lobular involution was calculated with reference to expected age-specific breast cancer incidence rates from the Iowa SEER registry. Compared to the Iowa SEER population, the relative risk (RR) of breast cancer was 1.88 (95% CI 1.59–2.21) for those with no involution, 1.47 (95% CI 1.30–1.51) for those with partial involution, and 0.91 (95% CI 0.75–1.10) for those with complete involution.

Lobular involution was associated with risk of breast cancer independently of other histological, reproductive, and demographic risk factors for the disease, but mammographic density was not among the risk factors considered. The extent of lobular involution modified risk in all of the subsets examined, most notably for women with cellular atypia on biopsy, where the RRs were 7.79 (95% CI 3.56–14.81) for those with no involution and 1.49 (95% CI 0.41–3.81) for those with complete involution.

## Mammographic density, involution, and ‘breast tissue age’

Mammographic density shares many of the features of ‘breast tissue age’ in the Pike model. Density is greatest at early ages, declines with increasing age, and is reduced by successive pregnancies and menopause (Boyd *et al*, 2002a). Similar to the concept of ‘breast tissue age’, mammographic density and the involution of breast epithelium and stroma are influenced by similar factors, including age, parity and menopause, and changes in these tissue components are reflected in changes in mammographic density. As suggested by the Pike model, cumulative exposure to mammographic density, breast lobules, and stroma that are responsible for radiological density may thus reflect cumulative exposure to hormones and growth factors that stimulate cell division in breast tissue and also be an important factor underlying the age-specific incidence of breast cancer.

Longitudinal studies of mammographic density support the concept that cumulative exposure is greater in those who develop breast cancer than in controls. Two longitudinal studies have examined the relationship between change in density and breast cancer risk, one over an average of 4–5 years (Maskarinec *et al*, 2006) and the other over an average of 7 years (Vachon *et al*, 2007a). Both used computer-assisted quantitative measurement of mammographic density and both showed that change in density with time was similar in women who developed breast cancer and those who remained free of disease (an example is shown in Figure 1B), but that at all ages, density was more extensive in women who develop breast cancer. Kerlikowske *et al* (2007), in a further longitudinal study reported that an increase in BI-RADS category in two mammograms at least 9 months and an average of 3 years apart was associated with a higher risk of breast cancer, and a decrease in category with a lower risk.

It is not clear at present to what extent these differences in results from longitudinal studies reflect differences in the menopausal status of the subjects studied, observer variation in the use of a qualitative method of assessing density, technical variations in mammography, or changes in the affected breast preceding the diagnosis of breast cancer.

With the exception of the study of Kerlikowske, the available evidence suggests that it is the extent of density at a given age, rather than the rate of change of density with increasing age, that is related to breast cancer risk (Maskarinec *et al*, 2006; Vachon *et al*, 2007a). However, there are important gaps in knowledge concerning cumulative exposure. We have little information about the factors that influence breast tissue characteristics at early ages, and it is also unclear whether interventions that reduce cumulative exposure will reduce the risk of breast cancer. Mammographic density and breast tissue involution are both associated with the risk of breast cancer independently of the other risk factors of age, parity, and menopausal status. It is not, however, known if the effects on breast cancer risk of mammographic density and breast tissue involution are independent of each other.

Reproductive and menstrual risk factors and body weight explain only 20–30% of the variation in density (Vachon *et al*, 2000). Twin studies have shown that genetic factors have a large function in explaining variation in mammographic density (Boyd *et al*, 2002b; Stone *et al*, 2006), and that after adjusting for age, parity, menopausal status, and body weight, an additive genetic model explained about 60% of the residual variance in percent density. The genetic factors that influence mammographic density may also explain variations in breast tissue involution.

## Limitations of existing studies of mammographic density, involution, and breast cancer risk

Our current understanding of the risks of breast cancer associated with both mammographic density and breast tissue involution may be underestimates. The risk of breast cancer associated with mammographic density is likely to be underestimated because of the limitations in all of the existing methods of measurement. None takes into account the thickness of the breast, and all are thus based on the projected area rather than the volume of breast tissue. Current computer-assisted methods of measurement require that a dichotomous threshold be placed between dense and non-dense tissue, and do not allow for a gradual change in tissue composition, as is likely to exist in reality (Boyd *et al*, 2005). Attempts to improve methods of measurement by addressing these and other limitations are in progress and may improve risk discrimination and strengthen a aetiological associations.

The risk of breast cancer associated with breast involution may also be underestimated. The data of Milanese *et al* are based necessarily on women who had biopsies. It seems likely that women in whom involution is most complete may also be less likely to have signs or symptoms that would prompt breast biopsy. Further, the definition of involution used by Milanese *et al* was based solely on the histology of breast lobules. As we show above, both the epithelium and stroma change with age, and both types of tissue may be relevant to carcinogenesis in the breast (Tlsty and Hein, 2001). Further, the use of quantitative methods to assess these tissues may provide more information than the three-category system used to date.

## Potential biological mechanisms

We have described in detail elsewhere a hypothesis, based on existing epidemiological evidence, to account for the association of mammographic density with the risk of breast cancer (Martin and Boyd, 2008), which we outline briefly here.

As we have discussed earlier, there is now extensive evidence that mammographic density is an independent risk factor for breast cancer that is associated with large relative and attributable risks for the disease. The epidemiology of mammographic density, including the influences of age, parity, and menopause, is consistent with it being a marker of susceptibility to breast cancer, similar to the concept of ‘breast tissue age’ described by the Pike model. Mammographic density reflects variations in the tissue composition of the breast. It is associated positively with collagen and epithelial and non-epithelial cells and negatively with fat. Mammographic density is influenced by some hormones and growth factors, as well as by several hormonal interventions. It is also associated with urinary levels of a mutagen. Twin studies have shown that most of the variation in mammographic density is explained by genetic factors.

The hypotheses that we have developed from these observations postulate that the combined effects of cell proliferation (mitogenesis) and genetic damage to proliferating cells by mutagens (mutagenesis) may underlie the increased risk of breast cancer associated with extensive mammographic density. There is clearly a need for an improved understanding of the specific factors that are involved in these processes and of the role played by the several breast tissue components that contribute to density. In particular, the identification of the genes and their biological functions, which are responsible for most of the variance in percent density, is likely to provide insights into the biology of the breast and may identify potential targets for preventive strategies for breast cancer. In the light of the other similarities with mammographic density described above, similar genetic factors may be responsible for the variations in breast tissue involution that are also associated with differences in the risk of breast cancer.

## Potential future directions

In this paper, we have proposed that the changes in mammographic density that occur with increasing age, parity, and menopause, as well as lobular involution in the breast, are closely related phenomena that both resemble Pike's theoretical concept of ‘breast tissue ageing’. Extensive mammographic density for a given age and delayed breast involution are both associated with an increased risk of breast cancer and are consistent with the hypotheses of the Pike model that the cumulative exposure of breast tissue to hormones and growth factors that stimulate cell division, as well as the accumulation of genetic damage in breast cells, are major determinants of breast cancer incidence.

There are, however, several gaps in our knowledge of these risk factors. Breast involution has to date been defined only in terms of breast lobules. The effects of extending the definition of involution beyond breast lobules to include stroma have not yet been examined. Both the epithelium and stroma are influenced by age, parity, and menopause, and stroma is known to have a function in the development and maintenance of the epithelium through paracrine growth stimuli and is thought to have a function in mammary carcinogenesis (Tlsty and Hein, 2001; Nelson and Bissell, 2006).

The relationship between mammographic density and histological definitions of involution in individuals, although suggested by the histological studies referred to above, has not yet been explicitly examined, and it is not known whether histological definitions of involution predict breast cancer risk independently of mammographic density.

It is quite likely that within the next few years the principal genetic variants that are associated with differences in mammographic density will be identified (Vachon *et al*, 2007b). These variants may help to identify biological pathways in the breast that are associated with variations in the epithelial and stromal tissues that participate in the process of involution. Some of these genes may also influence the risk of breast cancer.
